# The mediating role of food craving in the relationship between psychological distress and body mass index in adults: a cross-sectional analysis

**DOI:** 10.3389/fnut.2026.1872470

**Published:** 2026-06-18

**Authors:** Kadriye Toprak, Zeyneb Yildirim

**Affiliations:** Department of Nutrition and Dietetics, School of Health Sciences, Ankara Medipol University, Ankara, Türkiye

**Keywords:** anxiety, depression, excessive food craving, obesity, stress

## Abstract

**Background:**

The etiology of obesity involves complex interactions between psychological distress and negative eating behaviors. However, the specific mechanisms by which negative emotional states physiologically translate into weight gain remain unclear.

**Objective:**

The aim of this study is to examine the association of depression, anxiety, and stress with body mass index (BMI) in adults and to investigate the mediating role of food craving in these relationships.

**Methods:**

This cross-sectional study included 252 adults aged 19–65. The Depression, Anxiety, and Stress Scale (DASS-21) and the Food Craving Questionnaire (FCQ) were used, and anthropometric measurements were recorded. Mediation analyses were performed using the Hayes PROCESS macro (Model 4) with 5,000 bootstrap samples.

**Results:**

The study found positive correlations between all psychological symptoms, excessive food cravings, and BMI (*p* < 0.05). Participants who consumed a carbohydrate-and fat-rich diet had significantly higher FCQ scores compared to those who consumed a plant-based or protein-rich diet. Mediation analyses revealed that excessive food cravings played a significant partial mediating role in the relationship between BMI and depression (indirect effect: 19.4%), anxiety (indirect effect: 19.7%), and stress (indirect effect: 19.0%).

**Conclusion:**

In conclusion, our findings were consistent with a mediation model wherein psychological distress was associated with an increase in BMI both directly and indirectly through its link with elevated food cravings. A multidisciplinary approach that includes psychological interventions for emotion regulation and craving management, in addition to medical nutrition therapy, should be adopted for the treatment of obesity.

## 1 Introduction

Obesity is one of the major public health problems with an increasing prevalence worldwide. The World Health Organization (WHO) reports that 2.5 billion adults worldwide are overweight, and 890 million of them are obese. Obesity is associated with serious health problems such as type 2 diabetes, cardiovascular diseases, certain types of cancer, and metabolic syndrome, and it poses a significant burden on individual health and the economy (1). Obesity has a multifactorial etiology, and the formation of a positive energy balance between energy intake and expenditure is insufficient to explain the etiology of obesity. Research shows that complex relationships between psychological factors and neuroendocrine regulation play an important role in the development of obesity (2–4). In particular, psychological distress such as depression, anxiety, and stress is known to be strongly correlated with an increase in body mass index (BMI) (5–8). However, the exact physiological mechanisms linking psychological distress states to weight gain remain unclear.

The relationship between psychological symptoms and BMI increase is explained by various mechanisms, including neurobiological pathways, behavioral factors, and changes in eating habits, which may act alone or interact in complex ways. From a psychobiological perspective, chronic stress and negative mood lead to activation of the Hypothalamic–Pituitary–Adrenal (HPA) axis and increased cortisol release. It has been suggested that high cortisol levels stimulate the brain’s reward system (hedonic eating), independent of homeostatic hunger, creating an excessive desire (food craving) for energy-dense foods (2, 9). Various studies also show that excess glucocorticoids due to stress, illness, or medication use can be linked to increased consumption of high-energy foods (3, 4, 10–12). Sinha et al. (4) found that stress exposure, compared to a neutral state, was associated with increased craving and consumption of highly palatable foods, particularly in overweight or obese individuals. These results support the hypothesis that stress indirectly contributes to obesity by prompting individuals to turn to high-fat and sugary foods as a means of coping, relieving anxiety, or regulating stress, rather than directly slowing down metabolism (6, 7, 13).

In this mechanism, also referred to as emotional eating in the literature, excessive food craving has been investigated as a potential mediator of the relationship between psychological distress and obesity (2, 14). Food craving is defined as an intense desire to eat a specific food (15). Although not all food cravings are pathological, it has been noted that craving certain foods is associated with increased consumption of those foods and increased BMI, and this mediating role has been investigated (2, 14, 16, 17). Chao et al. (14) demonstrated that food cravings mediate the relationship between chronic stress and BMI. In a 6-month follow-up study by the same research group, they found that craving at the start of the study did not predict weight change at a statistically significant level but reported that it may take a long time for the effect of food craving on weight to become apparent (2). In contrast, Sinha et al. (4) stated that exposure to stress increases craving for highly palatable foods and, more importantly, that this increased craving level after stress predicts subsequent calorie intake. One study supporting this mediating role of excessive food craving is the “PLACE-19” study conducted on Polish adolescent girls. The study examined the association between stress and body weight and found that 21% of this relationship was mediated indirectly through “emotional eating” behavior. In other words, stress is associated with increased emotional eating, and this increased eating behavior may subsequently contribute to weight gain (6). These findings are consistent with a mediation model suggesting that the link between stress and eating behaviors is not solely direct but is instead statistically mediated by food cravings (motivation), which are further associated with actual food intake.

While mediators of emotional eating have been frequently studied in the current literature, the number of studies investigating the mediating role of excessive food cravings is insufficient. Furthermore, studies either focus solely on depression or address stress as a general umbrella concept. Studies that examine depression, anxiety, and stress (DASS-21 sub-dimensions) together and investigate the mediating role of excessive food cravings in their effect on BMI are limited. This study aims to examine the relationship between depression, anxiety, and stress levels and BMI, and the mediating role of excessive food cravings in this relationship from a behavioral and nutritional psychology perspective. The findings from this study are expected to contribute to multidisciplinary approaches that focus on emotional state, in addition to medical nutrition therapy, in the treatment of obesity.

## 2 Materials and methods

### 2.1 Study design and participants

This study is a descriptive cross-sectional study conducted with individuals who applied to fit Academy Nutrition Education and Counseling between October 2023 and November 2024. The study population consists of adults aged 19–65. The primary endpoint used for the sample size calculation was the relationship between excessive food cravings and BMI. The sample size of the study was calculated as a minimum of 57 using the G*Power 3.1.9.3 program, with a 95% confidence interval (*α* = 0.05) and 95% power (1-*β* = 0.95). The anticipated effect size was estimated based on the positive correlation between food craving scores and BMI reported in the validity study conducted by Muftuoglu et al. (18). The rationale for selecting these statistical parameters was to ensure high reliability and minimize Type I and Type II errors. However, because the current study aimed to test multiple psychological variables within mediation models, the sample was expanded to ensure sufficient statistical power. Methodological literature indicates that robust mediation analyses typically require larger samples to detect indirect effects reliably (19). Therefore, a final sample of 252 was necessary to obtain stable estimates for the proposed mediation models. To achieve this sample size, a total of 280 questionnaires were distributed to potential participants. Prior to statistical analysis, a rigorous data screening procedure was conducted to identify problematic questionnaires. After excluding surveys with missing or incomplete data from the primary scales, a final sample of 252 fully completed and valid questionnaires was retained for analysis, yielding an effective response rate of 90.0%.

A two-step data collection procedure was utilized. First, participants’ demographic characteristics and general lifestyle habits were assessed through face-to-face interviews. The scales related to psychological state and excessive food craving were completed by participants in a quiet room to avoid social desirability bias and protect their privacy. The inclusion criteria for the study were: being between the ages of 19 and 65, having no mental or cognitive problems that would prevent communication, and signing a voluntary consent form. Individuals with a diagnosis of major endocrinological disease (uncontrolled diabetes, hypo/hyperthyroidism, etc.) that could directly affect food intake, appetite, and body weight, pregnant women, nursing mothers, and individuals using psychotropic drugs (antidepressants, antipsychotics, etc.) known to have side effects on eating behavior were excluded from the study. Ethical approval for the study was obtained from the Non-Interventional Ethics Committee of Ankara Medipol University (AMÜ) on October 4, 2023 (Decision number: E-81477236-604.01.01-6787). Informed consent was obtained from all individual participants prior to their participation in the study.

### 2.2 Data collection form

Two scales were used in the study to assess individuals’ psychological states and food cravings, and the data collection tools were divided into three main sections, including these scales. The first section includes general information, including demographic, lifestyle, and anthropometric data; the second section consists of the Depression, Anxiety, and Stress Scale (DASS-21); and the third section consists of the Food Craving Questionnaire (FCQ).

#### 2.2.1 Demographic information and lifestyle habits

Individuals were asked about their gender, age, education level, physical activity status, and the food groups they predominantly consumed. Dietary patterns were assessed with a single multiple-choice question. To help participants accurately identify their habitual diet, each category included common food examples (e.g., bread, pasta, and pastries for carbohydrate-rich foods; meat and dairy for protein-rich foods; and vegetables and fruits for plant-based foods). While this approach allowed participants to self-select the pattern that best matched their daily intake, it relies on subjective self-reporting rather than quantitative nutritional measurement.

#### 2.2.2 Anthropometric measurements

Participants’ height and weight measurements were taken by researchers in accordance with standard procedures. BMI was used to determine body weight status. BMI was calculated by dividing body weight (kg) by height squared (m^2^). According to the World Health Organization classification, BMI < 18.5 kg/m^2^ is “underweight,” 18.5–24.9 kg/m^2^ “normal,” 25.0–29.9 kg/m^2^ “overweight”, 30.0–34.9 kg/m^2^ “obesity class I,” ≥35.0 kg/m^2^ “obesity class II” (20).

Depression, Anxiety, and Stress Scale (DASS-21): The DASS-21 scale was used to assess the psychological status of the participants. The scale consists of three subscales: depression, stress, and anxiety. It contains 21 items, with 7 questions per subscale. Each item is scored using a 4-point Likert scale (0 = not applicable to me, 1 = somewhat applicable to me, 2 = generally applicable to me, and 3 = completely applicable to me). Higher scores on the scale indicate greater symptom severity (21). In the Turkish validity and reliability study of the DASS-21, the internal consistency coefficients (Cronbach’s alpha) were reported as 0.87 for the depression subscale, 0.83 for the anxiety subscale, and 0.81 for the stress subscale (22). In the current study, the Cronbach’s alpha coefficients were calculated as 0.856 for the depression subscale, 0.832 for the anxiety subscale, and 0.772 for the stress subscale, indicating adequate to good reliability.

Food Craving Questionnaire (FCQ): One of the most widely used scales for assessing food cravings, the FCQ was developed by Cepeda-Benito et al. (23) to measure individuals’ food cravings. The Turkish validity and reliability study of the scale was conducted by Muftuoglu et al. (18). The scale consists of 39 items and is a 6-point Likert scale (never-always). There are no reverse-scored items in the scale. Responses to all items are summed to obtain a total score. Higher scores indicate more intense food cravings. The Turkish adaptation of the FCQ demonstrated high internal consistency, with a Cronbach’s alpha of 0.97 (18). In the present study, the Cronbach’s alpha coefficient for the FCQ was found to be 0.976, indicating high reliability for the current sample.

### 2.3 Statistical analysis

**Figure 1:**
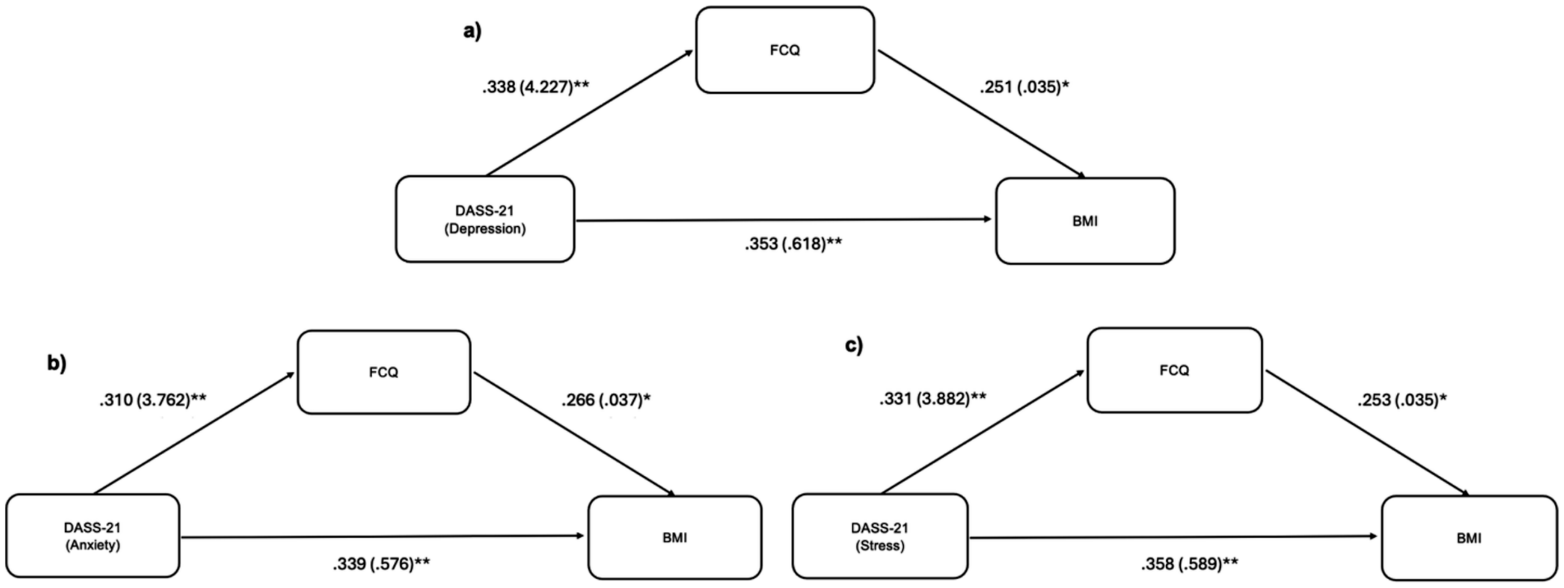
The mediation model (Model 4) with FCQ as a mediator between DASS-21 and BMI standardized coefficients are reported with unstandardized coefficients in brackets. **(a)** Mediation model for depression; **(b)** Mediation model for anxiety; **(c)** Mediation model for stress. **p < 0.001, *n* = 252.

The study’s data were analyzed using IBM SPSS Statistics v22. Variables such as age, gender, educational status, frequency of physical activity, and diet type, as well as scale scores, were examined using descriptive statistics. Continuous variables are presented as mean ± standard deviation (SD); categorical variables are presented as number and percentage (%). Prior to conducting inferential statistics, assumptions of normality, linearity, homoscedasticity, and multicollinearity were evaluated. Normality was verified using Skewness and Kurtosis values. Homoscedasticity was confirmed by visual inspection of the standardized residual scatterplots. Multicollinearity was checked via Variance Inflation Factor (VIF) and tolerance values, all of which were within acceptable limits. Pearson correlation analysis was applied to examine the relationships between variables. Additionally, the mediating effects among the DASS-21, FCQ, and BMI variables were analyzed using the PROCESS macro, following Hayes’ mediation analysis framework. To prevent suppression effects and multicollinearity among the highly correlated DASS subscales, separate and independent mediation models were constructed for depression, anxiety, and stress. To ensure the robustness of the findings, these mediation models were adjusted for covariates, including age, gender, educational status, and physical activity. Model 4 was used as the basis for the analyses, and the 95% confidence intervals (CIs) for the indirect effects were calculated using the 5,000 bootstrapping methods to determine the significance of the mediating effect. A confidence interval not containing the zero value was considered statistically significant (Figure 1) (24). The proportion of the mediated effect was calculated as the ratio of the indirect effect to the total effect [(indirect effect/total effect) × 100]. A significance level of *p* < 0.05 was accepted in all analyses.

## 3 Results

Table 1 presents the demographic information of the participants. 16.7% of the participants were male, and 83.3% were female, with an average age of 37.2. Most participants reported not engaging in any physical activity (54.0%). It was observed that the majority of participants reported a carbohydrate-heavy diet (43.7%).

**Table 1:** Demographic characteristics and dietary habits of the participants (*n* = 252).

Variables	Categories	M ± SD/*n* (%)
Age (years)		37.2 ± 11.96
Gender	Female	210 (83.3)
	Male	42 (16.7)
Education	Primary	1 (0.4)
	Secondary	3 (1.2)
	High school	28 (11.1)
	Undergraduate	182 (72.2)
	Postgraduate	38 (15.1)
Exercise frequency	Never	136 (54.0)
	1–2 times/mounth	24 (9.5)
	1–2 times/week	49 (19.4)
	3–4 times/week	27 (10.7)
	≥5 times/week	16 (6.3)
Self-perception of weight	Severely underweight	2 (0.8)
	Underweight	17 (6.7)
	Normal weight	119 (47.2)
	Overweight	81 (32.1)
	Obesity class I	23 (9.1)
	Obesity class II	10 (4.0)
Nutrition type	Carbohydrate-rich diet	110 (43.7)
	Fat-rich diet	28 (11.1)
	Protein-rich diet	75 (29.8)
	Plant-based diet	39 (15.5)
BMI (kg/m^2^)		25.6 ± 5.7
DASS-21 depression		8.5 ± 3.24
DASS-21 anxiety		7.8 ± 3.34
DASS-21 stress		9.5 ± 3.45
FCQ		110.9 ± 40.50

BMI, body mass index; DASS, depression anxiety stress scale; FCQ, food craving questionnaire.

The relationships between participants’ psychological symptoms (depression, anxiety, stress), excessive food cravings, and BMI values are presented in Table 2. Accordingly, positive correlations were observed between all values (*p* < 0.05).

**Table 2:** Correlation between psychological symptoms (depression, anxiety, stress), food craving and BMI (*n* = 252).

Variables	1	2	3	4	5
1. DASS depression	–				
2. DASS anxiety	0.729*	–			
3. DASS stress	0.741*	0.728*	–		
4. FCQ	0.332*	0.303*	0.334*	–	
5. BMI	0.407*	0.306*	0.373*	0.280*	–

DASS, depression anxiety stress scale; FCQ, food craving questionnaire; BMI, body mass index. *p < 0.001.

Data on the mediating relationship between the variables are presented in Figure 1 and Table 3.

**Table 3:** The mediating role of FCQ in the relationship between psychological symptoms (depression, anxiety, stress) and BMI (*n* = 252).

	Path	*β*	*B*	*SE*	*t/z*	*p*	95% CI
DASS depression							
*a*	DASS depression- > FCQ	0.338	4.227	0.809	5.222	<0.001	[2.584–5.763]
*b*	FCQ- > BMI	0.251	0.035	0.009	4.080	<0.001	[0.018–0.052]
*c*	DASS Depression- > BMI	0.438	0.766	0.090	8.489	<0.001	[0.589–0.943]
*c′*	DASS Depression- > BMI with FCQ as a mediator	0.353	0.618	0.109	5.643	<0.001	[0.415–0.846]
Indirect effect	DASS Depression- > FCQ- > BMI	0.085	-	0.029	2.894	0.004	[0.036–0.151]
DASS anxiety							
*a*	DASS anxiety- > FCQ	0.310	3.762	0.811	4.637	<0.001	[2.153–5.357]
*b*	FCQ- > BMI	0.266	0.037	0.009	3.940	<0.001	[0.018–0.055]
*c*	DASS anxiety- > BMI	0.422	0.716	0.089	8.075	<0.001	[0.479–0.964]
*c′*	DASS anxiety- > BMI with FCQ as a mediator	0.339	0.576	0.136	4.230	<0.001	[0.331–0.861]
Indirect effect	DASS anxiety- > FCQ- > BMI	0.083	–	0.028	2.924	0.003	[0.036–0.149]
DASS stress							
*a*	DASS stress- > FCQ	0.331	3.882	0.830	4.677	<0.001	[2.160–5.459]
*b*	FCQ- > BMI	0.253	0.035	0.010	3.730	<0.001	[0.017–0.053]
*c*	DASS stress- > BMI	0.442	0.726	0.085	8.528	<0.001	[0.506–0.954]
*c′*	DASS stress- > BMI with FCQ as a mediator	0.358	0.589	0.123	4.786	<0.001	[0.353–0.839]
Indirect Effect	DASS stress- > FCQ- > BMI	0.084	–	0.030	2.748	0.006	[0.034–0.156]

All mediation models were adjusted for covariates including age, gender, educational status, and physical activity. The 95% Confidence Intervals for the indirect effects were generated using 5,000 bootstrap resamples.

DASS, depression anxiety stress scale; FCQ, food craving questionnaire; BMI, body mass index.

Three different mediation models were established to examine the mediating role of excessive food cravings in the relationship between DASS-21 subscales and BMI. In each different model, where the DASS-21 depression, anxiety, and stress subscales were used as independent variables, respectively, the mediating role of excessive food craving in the associations of these variables on BMI was investigated. The results of each model are detailed in Table 3.

### 3.1 Model 1: [DASS-21 Depression] → [FCQ] → [BMI]

The direct effect of DASS-21 depression on BMI was found to be significant (*c’* path; *β* = 0.353, *SE* = 0.109, *t* = 5.643, *p* < 0.001). Furthermore, the indirect association mediated by FCQ accounted for 19.4% of the total effect (standardized indirect effect = 0.085, *p* = 0.004, 95% Bootstrapped CI [0.036, 0.151]).

### 3.2 Model 2: [DASS-21 Anxiety] → [FCQ] → [BMI]

The direct effect of DASS-21 anxiety on BMI was found to be significant (*c’* path; *β* = 0.339, *SE* = 0.136, *t* = 4.230, *p* < 0.001). Furthermore, the indirect association mediated by FCQ accounted for 19.7% of the total effect (standardized indirect effect = 0.083, *p* = 0.003, 95% Bootstrapped CI [0.036, 0.149]).

### 3.3 Model 3: [DASS-21 Stress] → [FCQ] → [BMI]

The direct effect of DASS-21 stress on BMI was found to be significant (*c’* path; *β* = 0.358, *SE* = 0.123, *t* = 4.786, *p* < 0.001). Furthermore, the indirect association mediated by FCQ accounted for 19.0% of the total effect (standardized indirect effect = 0.084, *p* = 0.006, 95% Bootstrapped CI [0.034, 0.156]).

Table 4 shows the scale scores by dietary type. While the excessive food craving scores of individuals with a carbohydrate-and fat-heavy diet were similar to each other, the scores were higher than the excessive food craving scores of individuals with a vegetable-and protein-heavy diet (*p* < 0.05). Although the DASS-21 subscale scores of individuals with a carbohydrate-and fat-heavy diet were statistically significant, they were generally higher than the scores of individuals with a vegetable-and protein-heavy diet (*p* > 0.05). The anxiety scores of individuals with a fat-heavy diet on the DASS-21 subscales were higher than those of the other groups (*p* < 0.05).

**Table 4:** Comparison of scale scores according to dietary patterns (*n* = 252).

Nutrition type	Plant-based	Protein-rich diet	Carbohydrate-rich diet	Fat-rich diet
FCQ	90.9 (38.62)^a^	98.5 (33.39)^a^	122.6 (39.44)^ab^	125.7 (45.01)^ab^
DASS-21 depression	7.7 (3.47)^a^	8.3 (3.05)^a^	8.8 (3.07)^a^	9.4 (3.86)^a^
DASS-21 anxiety	6.8 (3.68)^a^	7.7 (3.29)^a^	7.9 (3.28)^a^	8.8 (3.01)^b^
DASS-21 stress	9.1 (3.77)^a^	9.1 (3.07)^a^	9.7 (3.46)^a^	9.9 (3.92)^a^

DASS, depression anxiety stress scale; FCQ, food craving questionnaire. ^a,b^Means in the same row sharing the same superscript are not significantly different.

## 4 Discussion

This study investigated the mediating role of excessive food cravings in the relationship between psychological symptoms such as depression, anxiety, and stress and BMI in adults. The findings indicated that these psychological symptoms were positively associated with higher BMI and that excessive food cravings play a significant mediating role in these relationships. This study expands upon the existing literature by comprehensively evaluating the role of excessive food cravings across all three distinct psychological subdomains of the DASS-21 independently. This approach refines our understanding of how specific psychological symptoms are differentially associated with BMI.

The findings further indicated that depression, anxiety, and stress levels were positively associated with excessive food cravings and BMI. Additionally, excessive food cravings themselves were positively associated with BMI. All these findings indicate that negative mood, excessive food cravings, and weight gain are closely related. Our results are consistent with studies in the literature examining the relationships between negative psychological states and eating behaviors and body weight (14, 25, 26).

The relationship between depression, excessive food cravings, and high BMI may be explained by various neurobiological and behavioral mechanisms (27–29). In the current study, depression showed a strong correlation with excessive food cravings (*r* = 0.332, *p* < 0.001), and it has both a direct effect (*β* = 0.353, *p* < 0.001) and an indirect effect via excessive food craving (*β* = 0.085, *p* = 0.004) on BMI. It has been proposed that depression may be associated with increased food cravings through alterations in serotonergic signaling (27, 30). Some studies suggest that depressive symptoms may be associated with reduced serotonin activity, which could potentially increase preference for carbohydrate-rich foods. It has also been hypothesized that carbohydrate intake may transiently influence serotonergic pathways through tryptophan availability, potentially contributing to eating behaviors aimed at alleviating negative affect (31, 32). One study showed that obese individuals with depressive moods preferred foods high in energy and carbohydrates more than individuals in a neutral state (33). Similarly, another study reported that individuals with depressive symptoms consumed less fruit, vegetables, and legumes, but more sweets and refined sugars (34). In addition, the direct relationship between depression and BMI found in the current study was consistent with findings in the literature. It has been shown that depression can be associated with an increase in BMI through decreased physical activity, sleep disorders, and metabolic changes (35, 36).

The relationship between anxiety, overeating, and BMI increase may be interpreted through escape eating behavior and cognitive distraction mechanisms. According to emotion regulation theory, individuals may engage in overeating as a coping mechanism, to deal with negative emotions such as anxiety. It is stated that this behavior (anxiety-triggered overeating) occurs through escape eating behavior (37). Cognitive distraction theory also suggests that anxiety can contribute to overeating by distracting individuals from stressors and masking emotional distress with food consumption (38). One study concluded that participants with anxiety disorders had significantly higher emotional overeating scores compared to those without anxiety disorders, and that the severity of anxiety was positively related to emotional overeating (39). A similar study also showed that as anxiety levels increased among university students, their tendency toward emotional overeating also increased (40). Consistent with the literature, the current study also found that anxiety levels were positively correlated with excessive food cravings. In addition, it was found that anxiety and BMI are indirectly related through excessive food cravings and directly related. These findings are consistent with studies in the literature showing that anxiety is related to obesity (5, 8). Furthermore, previous literature has proposed that anxiety-related states may influence dopaminergic reward pathways, potentially increasing the preference for highly palatable foods rich in fat and sugar (41, 42). In the current study, the increased consumption of fat-rich foods by individuals with high anxiety levels may be compatible with these proposed mechanisms.

The study found that stress levels also showed positive correlations with both excessive food cravings and BMI, and that maladaptive eating tendencies played a partial mediating role in this relationship. These findings suggest that stress is associated with BMI not only directly but also indirectly through a link with increased eating urges. Several neurobiological and hormonal pathways have been hypothesized to underlie this relationship. Previous research has suggested that chronic stress may contribute to activation of the HPA axis and subsequent cortisol secretion. Elevated cortisol levels have been proposed to influence appetite regulation and reward-related eating behaviors, particularly increasing preference for energy-dense foods (43). In addition, cortisol-related metabolic alterations have been suggested to contribute to visceral fat accumulation and abdominal adiposity (44, 45). These biological processes are supported by various studies. For example, a cohort study showed that individuals with high hair cortisol concentrations were 72% more likely to be overweight or obese than those with low concentrations, and that this was positively correlated with increased BMI (46). A meta-analysis study showed that stress exposure diverts individuals away from healthy foods and toward energy-dense, fatty, and sugary foods (47). Furthermore, Kuckuck et al. (3) demonstrated that long-term biological stress in obese individuals significantly increases their tendency toward hedonic eating and excessive cravings for specific foods. Together, these biological and behavioral findings may help explain the observed associations between stress-related eating tendencies and increased BMI. Kim and Kim (48), in a 10-year longitudinal follow-up study, showed that psychosocial stress interacts with unhealthy eating behaviors (low dietary diversity and carbohydrate-heavy diet), increasing the risk of visceral fat accumulation and abdominal obesity in particular. In addition, the relationships between stress, reward-related eating behaviors, and obesity can also be explained by psychological coping. Various studies have shown that individuals may use eating as a coping strategy for stress (49–51). In the current study, the partial mediating role of these eating urges in the relationship between stress and BMI suggests that participants may lean toward eating behaviors as a maladaptive method of managing stress, which may contribute to a higher BMI.

In conclusion, the mediating effect observed in the present study is consistent with existing literature proposing that the psychophysiological burden associated with depression, anxiety, and stress may contribute to excessive food cravings as a coping-related or reward-oriented behavior associated with higher BMI. However, the neurobiological pathways discussed above should be interpreted as theoretically plausible mechanisms derived from prior literature rather than mechanisms directly demonstrated in the present study.

This study has some limitations. First, due to the study’s cross-sectional design, temporal directionality and causal relationships cannot be established. Consequently, it is not possible to definitively determine whether increased depression, anxiety, and stress precede or result from weight gain. Another limitation of the study is that the data were collected based on individuals’ self-reports. The fact that the data are based on subjective assessments may also introduce bias. As Evans et al. (52) noted, individuals’ statements regarding their psychological state and eating behavior may not fully reflect their actual psychological conditions. In terms of dietary assessment, intake was evaluated using broad categories with food examples rather than a validated quantitative tool such as a Food Frequency Questionnaire (FFQ) or a 24-h dietary recall. This subjective categorization limits the ability to evaluate precise macronutrient distributions. Furthermore, since biological markers related to the proposed neurobiological pathways (e.g., cortisol, serotonin, dopamine, or other HPA-axis-related biomarkers) were not measured, the present study was unable to directly evaluate or verify the underlying neurobiological mechanisms. Thus, the mechanistic explanations presented in the discussion should be interpreted cautiously as literature-based hypotheses rather than direct findings. Beyond these methodological constraints, the sample characteristics pose limitations to generalizability. The majority of the sample (83.3%) consisted of women, highlighting the need for future studies to investigate the role of gender differences in the relationship between psychological symptoms, food cravings, and obesity. Similarly, a vast majority of the participants (87.3%) possessed a bachelor’s degree or higher. Although educational status was adjusted for as a covariate in our mediation models to prevent statistical bias, this educational skew may still limit the extrapolation of the current findings to populations with more diverse educational backgrounds. Moreover, the generalizability of our findings is further constrained by potential selection and volunteer biases, as the study sample was recruited exclusively from a nutrition education and counseling setting. Individuals actively seeking professional nutrition counseling may differ substantially from the general population regarding their health awareness, dieting history, eating behaviors, weight-related concerns, and baseline psychological distress. These characteristics may have increased the likelihood of enrolling participants who were already more preoccupied with body weight, eating patterns, or psychological well-being, potentially influencing both the strength and direction of the observed associations. Therefore, the findings should be interpreted with caution when extrapolating to community-based or non-clinical populations. Finally, unmeasured potential confounders such as sleep quality, socioeconomic status, smoking, alcohol use, or a history of eating disorders could not be controlled for in the current study.

Despite the study’s limitations, it also has important strengths. First, the use of a mediation model enabled a more detailed analysis of the relationships among variables, rather than relying on simple correlations. This study contributes to the existing literature by suggesting that high levels of depression, anxiety, and stress may contribute to an increase in BMI through their positive association with excessive food cravings. Another strength of the study is its potential to identify individuals at risk for unhealthy eating behaviors and attitudes by assessing their levels of excessive food cravings. Finally, another strength of the study was its two-step data-collection approach. Face-to-face communication with participants during the data collection phase ensured sample reliability; however, psychological symptom and eating behavior scales requiring confidentiality were administered in an isolated environment independent of the researcher to prevent participants from feeling pressured. This method reduced the social desirability bias often encountered in sensitive questions, enabling participants to report their depression, anxiety, and excessive eating urges more reliably.

## 5 Conclusion

In conclusion, this study suggests that excessive food cravings play a significant mediating role in the association between psychological symptoms (depression, anxiety, stress) and higher BMI. The identification of a significant relationship between each psychological symptom and excessive food cravings and BMI highlights the importance of psychological factors in the etiology of obesity. The findings from this study emphasize the need to evaluate psychological factors and emotional eating behaviors in the prevention and treatment of obesity. In clinical practice, alongside nutrition counseling, implementing psychological interventions such as cognitive-behavioral therapy, stress management techniques, and developing emotion regulation skills may potentially contribute to more effective and sustainable outcomes in obesity management. Future research should use experimental and longitudinal designs to deepen understanding of the mechanisms underlying these relationships, conduct validation studies across diverse populations, and assess the effectiveness of psychological interventions.

## Data Availability

The raw data supporting the conclusions of this article will be made available by the authors, without undue reservation.
